# Abdominal obesity is a common finding in normal and overweight subjects of Chile and is associated with increased frequency of cardiometabolic risk factors

**DOI:** 10.1371/journal.pone.0194644

**Published:** 2018-03-26

**Authors:** Beatriz Villanueva, Antonio Arteaga, Alberto Maiz, Víctor A. Cortés

**Affiliations:** Departmento de Nutrición, Diabetes y Metabolismo, Escuela de Medicina, Pontificia Universidad Católica de Chile, Santiago, Chile; CUNY, UNITED STATES

## Abstract

**Background/Objectives:**

Abdominal obesity (AO) is associated with elevated risk for cardiovascular diseases; however, this association is less clear for non-obese people. We estimated the association of AO and cardiovascular risk factors (CVRF) and disease in non-obese adult individuals from Chile.

**Subjects/Methods:**

5248 adults (15 years of age or older) of both sexes from the Chilean National Health Survey (October 2009 –September 2010, response rate 85%.) were included. Information on myocardial infarction and stroke was self-reported. BMI, waist circumference (WC), arterial pressure, plasma glucose, and cholesterol levels were measured. Predictive accuracy of WC was evaluated by area under curve of receiver operating characteristic analysis and cut off points were established by Youden Index. Relationship between AO and CVRF was analyzed by Chi-squared tests.

**Results:**

Normal weight/overweight/obesity were present in 34.4%/45.2%/18.1% of men and 33.4%/33.6%/27.5% of women. Predictive accuracy of WC to identify at least one CVRF was 0.70/0.67 and optimal cutoff points for WC in non-obese subjects were 91/83 cm in men/women, respectively. AO was present in 98.2%/99.1% of obese, 70.5%/77.4% of overweight and 12.4%/16.4% of normal weight men/women. AO was associated with increased frequency of CVRF in overweight men (6/8 and stroke) and women (4/8) and higher frequency in normal weight men (8/8 and myocardial infarction/stroke) and women (6/8 and myocardial infarction).

**Conclusions:**

WC cutoff points calculated for non-obese chilean population discriminate more differences in CVRF in normal weight woman. AO significantly increases the frequency of CVRF and diseases in overweight and especially normal weight individuals. WC can be used as a low cost, feasible and reproducible predictor for CVRF in non-obese individuals in most clinical settings.

## Introduction

Excessive adiposity is a risk factor for cardiovascular diseases and type 2 diabetes mellitus. Prospective studies have shown that obese and overweight individuals [1–3] as well as those with excessive abdominal fat [4–6], have increased risk for cardiovascular diseases and death, indicating a possible causative role of excessive abdominal adiposity in these conditions [7].

Numerous methodologies to study body composition and adipose tissue distribution are currently available; however, many of them cannot be used in usual clinical settings. For that reason, anthropometric approaches that require few resources and have acceptable correlation with reference techniques have been developed [8].

Expert groups have recommended waist circumference (WC) as an anthropometric approximation for intra-abdominal fat content. In fact, WC has a good direct correlation with more direct methods for quantifying body composition and is a technically and economically accessible procedure in a variety of clinical scenarios, including those in developing countries or rural clinics [8, 9].

The World Health Organization (WHO) [10], American Association of Endocrinology (AAE), and US National Program for Cholesterol Education (NCEP ATP III) [11] proposed to define AO with mean WC of adults with a body mass index (BMI) of 30 kg/m^2^. Therefore, WC cutoffs defined by these organizations were 88 cm for women and 102 cm for men. By contrast, the International Diabetes Federation (IDF) proposed to use average WC of subjects with a BMI of 25 kg/m^2^ and recognized that ethnical differences must be considered to define correlation between AO and metabolic syndrome risk factors [12]. Consequently, IDF did not make specific cutoff recommendations for Hispanic ethnicity but suggested to use values of South Asian population (80 cm for women and 90 cm for men).

Herein we aimed to determine the predictive capability of WC for cardiovascular risk factors (CVRF) and estimate optimal WC cutoff values for the hispanic population of Chile. We evaluated the association between abdominal obesity and CVRF and diseases in non-obese individuals.

## Subjects and methods

### 1. General design and measurements

This cross-sectional study was a secondary analysis of the National Health Survey of Chile (ENS) 2009–2010. General methodology of ENS is detailed elsewhere [13].

Participants were evaluated by research nurses who determined arterial blood pressure and anthropometric parameters. Blood aliquots were drawn to determine fasting glycemia, total cholesterol, low density lipoprotein cholesterol, high density lipoprotein cholesterol and triglycerides.

Trained interviewers collected data on demographics and physician-diagnosed cardiovascular diseases (myocardial infarction and stroke) as well as other medical conditions not considered.

Height and weight were measured using stadiometers and digital scales. BMI was calculated by the regular formula (weight [kg]/height [m^2^]. WC was quantified in the mid axillary line at the midpoint between the lower costal ridge and the upper margin of superior iliac crest, using a flexible plastic tape that was replaced after 80 determinations.

Systolic and diastolic arterial blood pressure was determined with an automated device (OMRON-HEM 713 C). These determinations were performed in the morning after overnight fasting and bladder emptying and 5 minutes of rest in a chair. Two separate measurements were taken for 2 minutes and average of both values was used for analysis.

### 2. Definition of the variables

A) BMI categories: categories of normal weight, overweight and obese were defined according to BMI ranges of 18.5–24.9 kg/m^2^, 25–29.9 kg/m^2^ and 30–39.9 kg/m^2^, respectively.
B) Systolic hypertension: systolic arterial pressure ≥ 140 mmHg or self-report.
C) Diastolic hypertension: diastolic arterial pressure ≥ 90 mmHg or self-report.
D) Total hypercholesterolemia: total cholesterol (total-C) ≥ 200 mg/dl.
E) LDL hypercholesterolemia: LDL cholesterol (LDL-C) ≥ 130 mg/dl.
F) Decreased HDL cholesterol: HDL cholesterol (HDL-C) ≤ 40 mg/dl in men or ≤ 50 mg/dl in women.
G) Hypertriglyceridemia: triglycerides ≥ 150 mg/dl.
H) Impaired fasting glucose: fasting blood glucose between 100 and 125 mg/dl.
I) Diabetes mellitus: fasting blood glucose ≥ 126 mg/dl or self-report.
J) CVRF: WHO guidelines establish that development of WC cutoff points should identify at least one of the three following risk factors: high blood pressure, elevated cholesterol or elevated blood glucose [14]. Our model identifies at least one of the following risk factors: systolic or diastolic hypertension, total hypercholesterolemia, LDL hypercholesterolemia, decreased HDL-C hypertriglyceridemia, diabetes mellitus or impaired fasting glucose.
K) Abdominal obesity (AO): in this work, we used two set of values determined as indicated in the following section. For all BMI ranges, WC cutoffs for AO definition were ≥ 88 cm in women and ≥ 93 cm in men. For individuals with BMI in normal and overweight range, WC cutoffs were ≥ 83 cm in women and ≥ 91 cm in men.

### 3. Statistical analysis

A total of 5293 subjects of both sexes and older than 15 years of age composed the total sample of the ENS 2009–2010. In the ENS 2009–2010 some determinations were not complete for individual subjects. These omissions are randomly distributed across the studied groups and thus in some tables the totals do not correspond to the simple sum of individual groups. Subjects with mistaken or rejected measurement of waist circumference were excluded from analysis, leaving a final sample of 5248 subjects.

Expansion factors specifically designed for this survey were used to give each participant the weight that corresponds according to the complex sample design and to correct the distortion of the raw sample, making it coincident with the census population projection to January 2010 for Chilean adults over 15 years of age. A detailed mathematical method for this procedure was published elsewhere [13].

Confounding factors for the association between WC and CVRF were determined by logistic regression analysis.

A predictive model was generated using the dependent variable CVRF and the independent variable WC for each sex. Predictive accuracy of WC was evaluated by area under curve of receiver operating characteristic (AUC-ROC) analysis [15]. Calibration of the model was determined using the Hosmer-Lemeshow goodness-of-fit test and was considered adequate with p > 0.05, indicating that the observed probability of having cardiovascular risk factors do not significantly differ from the probability predicted by the model [16]. Youden Index was calculated (highest sensitivity + specificity—1) to identify the best cutoff value based on the largest vertical distance between the ROC curve and the diagonal curve (or maximal value).

Categorical variables are presented as frequency according to individual estimated sample of each variable and percentage; their association with AO was analyzed with the independent χ^2^ statistic in a contingency model of exposure or non-exposure. Fisher´s exact test was used when the expected frequency was below 5. Differences were considered significant with a p value <0.05. Numerical variables are presented as mean and confidence intervals.

Scatter plots were generated to analyze the relationship between WC and BMI in both sexes. Spearman correlation was used to determine the association between these variables.

Stata SE software (version 13, College Station, Texas, USA) was used for statistical analysis and graphs.

## Results

To study cardiometabolic impact of AO we analyzed the latest National Health Survey of Chile (ENS 2009–2010). Table 1 shows the sample characteristics. In total, 5248 persons aged 15 years or older were included in the present analysis. Mean age was 40.5 years and 42.3 years in men and women, respectively. Overweight was the most frequent BMI category in both sexes. About one third of men and one fourth of women had hypertension. The most frequent CVRF was total hypercholesterolemia. Diabetes Mellitus was present with more frequency in women and stroke with more frequency in men.

**Table 1:** General characteristics of the sample.

	Men		Women	
Number of cases	2132		3116	
Age (years, mean—CI)	40.5 (39.2–41.7)		42.3 (41.3–43.3)	
BMI categories	N	%	N	%
Normal weight (18.5–24.9 kg/m^2^)	645	34.42	938	33.41
Overweight (25–29.9 kg/m^2^)	851	45.17	942	33.56
Obese (30–39.9 kg/m^2^)	341	18.07	771	27.47
**Conditions**				
Systolic hypertension	400	21.15	477	16.92
Diastolic hypertension	273	14.50	195	6.92
Total hypercholesterolemia	430	38.99	601	38.09
LDL hypercholesterolemia	335	30.56	473	30.00
Decreased HDL-C	459	24.14	913	32.17
Hypertriglyceridemia	397	35.97	429	27.17
Impaired fasting glucose	235	12.38	288	10.13
Diabetes Mellitus	146	7.91	260	9.51
AMI	64	3.37	87	3.06
Stroke	48	2.54	50	1.76

Number of observations were calculated based on percentages and total observations for each variable in survey data. CI: confidence interval. HDL-C-: concentration of cholesterol in high-density lipoproteins; AMI: acute myocardial infarction.

To assess the role of WC as an independent predictor of cardiovascular risk, we first performed logistic regression for confounding factors in the whole sample, i.e., including individuals of all the BMI ranges and both sexes. We found that age, height, educational level, physical activity, smoking status and alcohol did not influence the relationship between WC and CVRF. In this global analysis of the sample, the mean BMI was 27.2 kg/m^2^.

**Fig 1:**
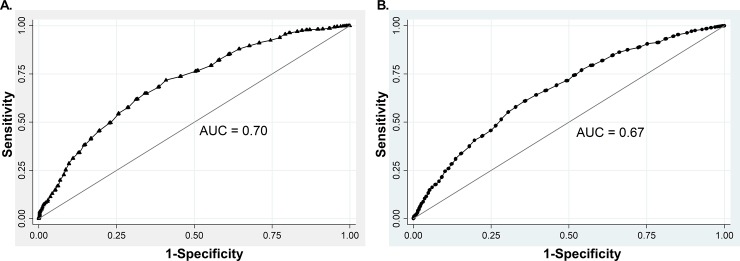
ROC analysis for WC and CVRF in the whole sample defined by the presence of at least one of the following risk factors: Systolic or diastolic hypertension, total hypercholesterolemia, LDL hypercholesterolemia, decreased HDL-C hypertriglyceridemia, impaired fasting glucose or diabetes mellitus in men (A) and women (B).

Next, we generated a model for CVRF prediction using WC as an independent variable in the whole sample. AUC-ROC value was 0.70 for men (Fig 1A) and 0.67 for woman (Fig 1B). These models showed good fitness as indicated by Hosmer-Lemeshow values of 15.32 (p = 0.0533) and 13.84 (p = 0.0860) for men and women, respectively. To determine the WC cutoff value for the definition of AO, we used Youden index analysis. In men, a WC cutoff value of 93 cm had Youden index of 0.31 for CVRF prediction and 64.9% sensibility, 66% specificity, and 65.2% of correctly classified subjects. In women, a WC cutoff value of 88 cm had a Youden index of 0.25 and 64.08% sensibility, 60.63% specificity, and 62.84% of correctly classified subjects.

Using 93 cm and 88 cm as cutoff values for AO in men and women, respectively, we analyzed the relationships between AO and the frequency of cardiometabolic risk factors in overweight and normal weight individuals. As shown in Table 2, AO was associated with increased frequency of systolic and diastolic hypertension, total and LDL hypercholesterolemia, Impaired fasting glucose and diabetes mellitus in overweight men. In overweight women, AO was associated with systolic hypertension, hypertriglyceridemia, impaired fasting glucose and diabetes mellitus. In both sexes, AO was associated with increased frequency of stroke.

**Table 2:** Frequency of CVRF, AMI and stroke in overweight individuals (BMI: 25–29.99 kg/m^2^) with AO.

	Men (n = 851)					Women (n = 942)				
	AO (-)		AO (+)			AO (-)		AO (+)		
	N	%	N	%	P	N	%	N	%	P
Number of cases	321	37.7	530	62.3		394	41.8	548	58.2	
Systolic hypertension	24	7.64	148	27.93	<0.0001	36	8.44	128	21.63	<0.0001
Diastolic hypertension	34	10.51	92	17.39	0.016	25	5.79	41	6.97	0.109
Total hypercholesterolemia	60	34.24	155	50.64	0.026	79	33.85	135	44.31	0.096
LDL hypercholesterolemia	49	28.53	128	41.99	0.044	54	23.47	123	40.49	0.125
Decreased HDL-C	49	15.36	127	23.61	0.083	134	31.43	166	28.03	0.821
Hypertriglyceridemia	57	32.64	120	38.96	0.061	39	16.71	85	27.77	0.015
Impaired fasting glucose	17	5.17	88	16.45	<0.0001	22	5.09	89	15.05	<0.0001
Diabetes Mellitus	12	3.63	46	8.97	0.001	12	2.94	83	14.47	<0.001
AMI	8	2.57	18	3.36	0.307	11	2.59	19	3.28	0.222
Stroke	2	0.73	25	4.72	0.019	6	1.38	17	2.95	0.001

AO: Abdominal obesity defined by WC cutoff of 93 cm in men and 88 cm in women. Other abbreviations as in Table 1.

The implications of AO on cardiometabolic health of normal weight individuals are shown in Table 3. In normal weight men, the association between AO and CVRF and disease was even higher than in overweight subjects, since the frequency of AMI and stroke as well as all CVRF analyzed (8 over 8) were increased. By contrast, in normal weight women, AO was associated with an increased frequency of only 3 over 8 CVRF (total hypercholesterolemia, hypertriglyceridemia and impaired fasting glucose) and AMI.

**Table 3:** Frequency of CVRF, AMI and stroke in normal weight individuals (BMI:18.5–24.99 kg/m^2^) with AO.

	Men (n = 645)					Women (n = 938)				
	AO (-)		AO (+)			AO (-)		AO (+)		
	N	%	N	%	P	N	%	N	%	P
Number of cases	584	90.5	61	9.5		873	93.1	65	6.9	
Systolic hypertension	59	11.59	10	19.89	< 0.0001	76	10.18	12	23.54	0.113
Diastolic hypertension	28	5.47	12	22.81	< 0.0001	21	2.77	2	3.03	0.06
Total hypercholesterolemia	67	22.33	24	79	0.034	108	25.11	20	56.24	0.002
LDL hypercholesterolemia	47	15.41	24	78.46	0.024	83	19.37	14	38.35	0.22
Decreased HDL-C	85	16.71	35	67.01	< 0.0001	191	25.55	5	10.02	0.769
Hypertriglyceridemia	48	15.99	18	57.51	<0.0001	46	10.57	8	22.29	0.001
Impaired fasting glucose	39	7.58	7	13.06	<0.0001	25	3.39	3	5.83	0.001
Diabetes Mellitus	17	3.37	10	20.39	<0.0001	31	4.23	3	5.72	0.299
AMI	5	1.07	2	3.86	0.007	15	1.97	2	2.85	0.04
Stroke	2	0.32	1	0.13	0.01	5	0.61	0	0	0.515

AO: Abdominal obesity defined by WC cutoff of 93 cm in men and 88 cm in women. Other Abbreviations as in Table 1.

Because of the lower number of associations between WC and CVRF in normal weight versus overweight women, we decided to investigate whether the cause of this apparent lower discriminative power was an inadequate WC cutoff point for this subgroup of individuals. For this purpose, we performed a new ROC analysis that exclusively included normal and overweight subsample in both sexes. In this new analysis, the mean BMI was 25.1 kg/m^2^.

**Fig 2:**
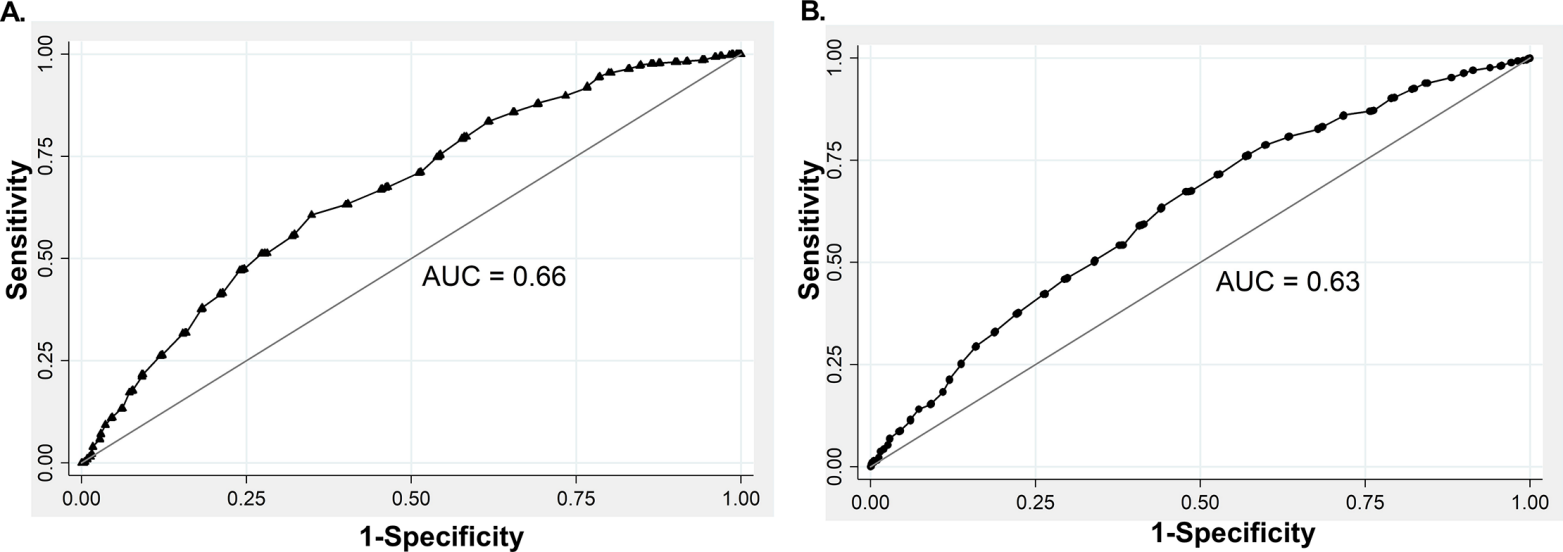
ROC analysis for WC and CVRF in normal and overweight subsample defined by the presence of at least one of the following risk factors: Systolic or diastolic hypertension, total hypercholesterolemia, LDL hypercholesterolemia, decreased HDL-C hypertriglyceridemia, impaired fasting glucose or diabetes mellitus in men (A) and women (B).

As shown in Fig 2, AUC-ROC for normal weight and overweight subjects was 0.66 and 0.63, for men and woman, respectively. These models also showed good fitness as indicated by Hosmer-Lemeshow values of 11.18 (p = 0.1919) and 9.93 (p = 0.2696) for men and women, respectively. We found that in normal and overweight men a WC cutoff value of 91 cm had a Youden index of 0.26 for CVRF prediction and 60.66% sensibility, 65.11% specificity, and 62.43% of correctly classified subjects. In normal and overweight women, a WC cutoff value of 83 cm had Youden index of 0.19 and 63.02% sensibility, 55.99% specificity, and 60.01% of correctly classified subjects.

With these new WC cutoff values (91 cm in men and 83 cm in women) we re-evaluated the association between AO and the frequency of CRVF in overweight and normal weight individuals, excluding underweight and obese subjects. As shown in Table 4, AO was associated with equivalent increased frequency of CVRF in overweight men and women (compare with Table 2).

**Table 4:** Frequency of CVRF, AMI and stroke in overweight individuals (BMI: 25–29.99 kg/m^2^) with AO defined by lower WC cutoff values.

	Men (n = 851)					Women (n = 942)				
	AO (-)		AO (+)			AO (-)		AO (+)		
	N	%	N	%	P	N	%	N	%	P
Number of cases	221	26	630	74		166	17.6	776	82.4	
Systolic hypertension	15	6.85	156	24.73	<0.0001	8	4.55	153	18.38	<0.0001
Diastolic hypertension	11	5.07	117	18.52	0.019	7	4.02	59	7.11	0.777
Total hypercholesterolemia	39	33.65	174	47.89	0.014	39	36.66	173	40.14	0.544
LDL hypercholesterolemia	30	25.82	149	41.06	0.002	22	20.41	156	36.26	0.169
Decreased HDL-C	35	15.75	139	21.75	0.298	60	33.47	240	28.63	0.636
Hypertriglyceridemia	36	30.83	139	38.26	0.16	16	15.31	107	24.8	0.016
Impaired fasting glucose	13	5.96	91	14.18	0.01	9	4.93	96	11.54	0.015
Diabetes Mellitus	7	3.07	26	8.26	0.003	6	3.33	84	10.44	0.009
AMI	6	2.57	21	3.23	0.321	4	2.34	26	3.13	0.271
Stroke	1	0.61	26	4.08	0.015	3	0.19	18	2.18	0.193

AO: Abdominal obesity defined by WC cutoff of 91 cm in men and 83 cm in women. Other abbreviations as in Table 1.

In normal weight men, AO continued to be associated with increased frequency of all CVRF (8 over 8), AMI and stroke (Table 5, compare with Table 3). Interestingly, the new WC cutoff value for non-obese women (83 cm instead of 88 cm), determined a higher frequency of CVRF (6 over 8: systolic and diastolic hypertension, total and LDL hypercholesterolemia, hypertriglyceridemia and impaired fasting glucose) and AMI in normal weight women (Table 5, compare with Table 3). These results suggest that lower WC cutoff values are required to correctly classify AO in normal weight women, in terms of their CVFR and disease frequency.

**Table 5:** Frequency of CVRF, AMI and stroke in normal weight individuals (BMI:18.5–24.99 kg/m^2^) with AO defined by lower WC cutoff values.

	Men (n = 645)					Women (n = 938)				
	AO (-)		AO (+)			AO (-)		AO (+)		
	N	%	N	%	P	N	%	N	%	P
Number of cases	557	86.3	88	13.7		747	79.6	191	20.4	
Systolic hypertension	53	10.9	17	23.57	< 0.0001	45	7.08	50	30.9	<0.0001
Diastolic hypertension	27	5.48	15	20.22	< 0.0001	16	2.42	7	4.63	0.014
Total hypercholesterolemia	63	21.69	32	73.73	0.005	92	24.67	40	41.72	<0.0001
LDL hypercholesterolemia	43	14.8	32	72.08	0.003	71	19.15	28	29.22	0.006
Decreased HDL-C	80	16.39	46	61.85	< 0.0001	171	26.66	23	14.19	0.683
Hypertriglyceridemia	46	16.04	22	50.81	<0.0001	32	8.73	25	26.75	0.001
Impaired fasting glucose	36	7.38	10	13.62	<0.0001	17	2.72	12	7.7	<0.0001
Diabetes Mellitus	15	3.19	13	19.41	<0.0001	21	3.37	14	9.07	0.062
AMI	5	0.97	3	4.19	0.003	3	0.47	16	10	0.004
Stroke	2	0.32	1	1.24	0.006	4	0.61	1	0.36	0.665

AO: Abdominal obesity defined by WC cutoff of 91 cm in men and 83 cm in women. Other abbreviations as in Table 1.

**Fig 3:**
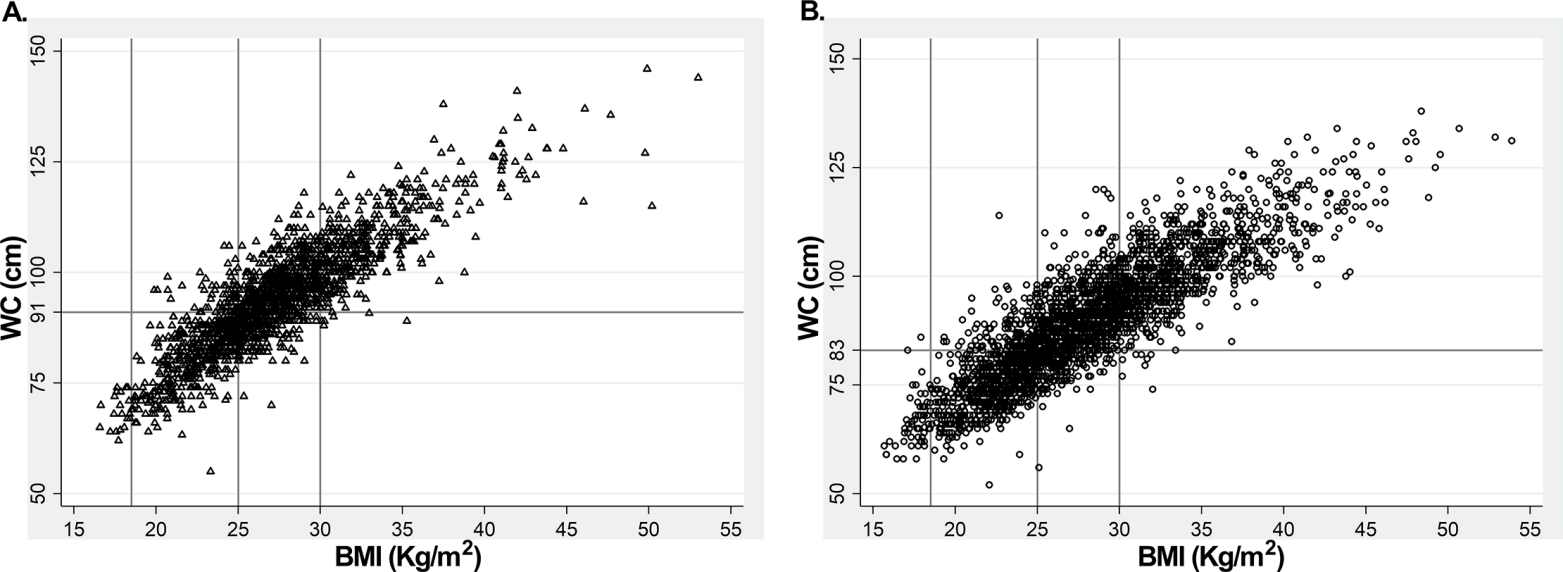
Correlation between BMI and WC in adults from ENS-Chile 2009–2010. Anthropometric values were measured in a total of 2132 men (A) and 3116 women (B). Vertical lines indicate the BMI values defining normal weight (18.5–24.9 kg/m^2^), overweight (25–29.9 kg/m^2^) and obesity (≥ 30 kg/m^2^). Horizontal lines indicate the WC cutoff defining AO in men (91 cm) and women (83 cm).

Lastly, we analyzed the relationship between BMI and WC; and the frequency of AO in normal and overweight population defined by the WC cutoff points found herein (91 and 83 cm for men and women, respectively) (Fig 3). As expected, WC was directly correlated with BMI in both sexes (men, r = 0.86, p <0.00001; women, r = 0.87, p <0.00001) and virtually all obese individuals had AO (98.2% and 99.1%, men and women, respectively). Notably, a high proportion of overweight and normal weight subjects also presented AO (70.5%/77.4%, and 12.4%/16.4% of overweight and normal weight men and women, respectively, had AO). Since almost all obese subjects had AO, these results indicate that the clinical emphasis of excessive WC, and thus intra-abdominal adiposity, on cardiovascular health must be focused in normal and overweight non-obese individuals.

## Discussion

Obesity is recognized as a major cardiovascular risk factor. The World Health Organization and the US National Institutes of Health (NIH) have proposed, based on prospective mortality studies, to use BMI as the "indicator" to estimate the cardio-metabolic risk magnitude. In fact, BMI levels ≥ 30 kg/m^2^ define the diagnosis of obesity, which is associated with the highest cardio-metabolic risk compared to other BMI categories [10]. Nonetheless, elevated WC further increases the cardio-metabolic risk determined by elevated BMI [17–19]. Therefore, some guidelines consider WC as a risk estimator with added value to the BMI [20, 21].

Concordantly, whereas some multivariate models have found that global adiposity assessed by the BMI is not associated with CVD risk, indicators of visceral adiposity, such as WC and waist to hip ratio (WHR), are directly associated with increased risk of CVD and all-cause mortality [22].We chose to use WC instead of WHR because the latter is not able to reliably predict the absolute amount of visceral adipose tissue that is also related to total adiposity [23]; and instead of waist to height ratio because no formal cutoff values have been established by international clinical guidelines for adults yet, in spite of the increasing studies emphasizing its clinical utility and diagnostic accuracy [24].

Our results support that WC behaves as an independent and statistically acceptable discriminator of CVRF frequency in the Hispanic population of Chile. ROC analysis of the whole sample, including all BMI ranges, showed an estimated predictability of 0.70 in men and 0.67 in woman, for identifying at least one cardiovascular risk factor. Our results are similar to others previously reported for the Chilean population [25]

Our study showed that in non-obese men, WC cutoffs for AO based on AUC-ROC analysis with either all categories of BMI (93 cm) or restricted to normal and overweight subjects (91 cm), was strongly associated with increased frequency of most or all CVRF and diseases in overweight and normal weight individuals.

In contrast, we noted that the frequency of CVRF in non-obese women was significantly different with the two different AO definitions reported herein. When AO was defined by AUC-ROC analysis based on the whole sample (88 cm), normal weight women had 3 over 8 CVRF; whereas, when AO was defined by AUC-ROC analysis of non-obese women exclusively (83 cm) they had 6 over 8 CVRF. This suggests that the cutoff point established by the whole sample may be inadequate for the subpopulation of non-obese women. The causes for this finding are yet to be elucidated; however, it could partially be explained by alterations in inflammatory [26] and oxidative stress [27] status induced by the shift from gynoid to android adiposity phenotype, further increasing the proportional risk for cardiovascular diseases in women in comparison with men. Our study is unable to elucidate causality relationships, because of its descriptive nature and cross-sectional design. Future prospective experimental or quasi experimental studies will be required to answer these pending questions.

In our study, we found that AO is associated with a higher deleterious impact on the frequency of CVRF in normal weight in comparison with overweight individuals in both sexes. Since WC was found to be directly correlated with BMI in both sexes and virtually all obese individuals had AO, we contend that WC determination is impractical for this type of subjects. In fact, NIH guidelines for the identification, evaluation and treatment of overweight and obesity in adults indicate that WC measurement is particularly useful in patients who are categorized as normal or overweight and that in individuals with BMI of ≥ 35 kg/m^2^, WC cutoff points lose incremental predictive power of the disease risk classification of BMI [11]. IDF guidelines state that in individuals with BMI 30 kg/m^2^, central obesity can be assumed, and WC does not need to be measured for practical clinical purposes [12].

The cross-sectional design of our study precludes the generation of definitive cutoff points for the definition of AO. However, we propose WC limits of 91 cm for men and 83 cm for women, as a suitable approximation for normal and overweight Hispanic individuals of Chile, since they allow the prediction of at least one CVRF. Importantly, our proposed cutoff points for non-obese individuals were calculated in a subsample with a mean BMI of 25.1 kg/m^2^, similar to the one reported in the Southeast Asian population used for establishing the IDF definition of AO [28] and proposed to be used in ethnic Central and South American population [29].

Overall, AO was present in an unexpectedly high proportion of overweight and normal weight people and it was associated with elevated frequency of cardiovascular risk factors and diseases. This finding stresses the importance of incorporating AO as a cardiometabolic risk estimator in normal and overweight individuals, in whom BMI clearly underestimates cardiovascular risk.

Considered together, our results support the use of WC at the clinical level to refine cardiovascular risk assessment. The low cost, feasibility and reproducibility of WC determination strongly advocates for its use [30]. Furthermore, since quantitative determination of intra-abdominal adiposity is unfeasible in most clinical settings, WC is an acceptable surrogate indicator [30]. Previous studies, using various designs and cutoff criteria, have reached similar conclusions across different populations worldwide [31–34].

Advances in the knowledge of adipose tissue biology have shed light on the mechanisms by which obesity and abnormal body fat distribution promotes atherosclerosis, dysglycemia, dyslipidemia and hypertension, including the ability of adipose tissue to modulate ectopic lipid accumulation in insulin responsive tissues [35], the secretion of protein and lipid endocrine mediators (adipokines) [36] and the modulation of tissue specific and general inflammatory status [37].

The main the limitations of our study are its cross-sectional design that prevents causation analysis, and the use of self-report as indicator of coronary and cerebrovascular disease.

In summary, we found that: 1) WC and BMI are directly correlated in both sexes and that the diagnosis of body adiposity, based on these indicators, is highly concordant for people with BMI > 30 kg/m^2^. By contrast, important proportions of non-obese people (BMI 18.5–29.9 kg/m^2^) have AO, indicating that, in these people, the sole analysis of BMI may underestimate their cardiometabolic risk. 2) WC cutoff points of 91 cm for men and 83 cm for woman allow to discriminate at least one CVRF in normal and overweight adult Hispanic population of Chile.

We conclude that WC is a useful, inexpensive and easy to use tool for predicting CVRF and its use must be promoted in normal and overweight individuals in whom its incrementing predictive power to detect disease risk classification remains valid.

## Abbreviations

AO: Abdominal obesity
WC: Waist circumference
CVRF: Cardiovascular risk factor
AUC-ROC: Area under curve of receiver operating characteristic
AMI: Acute myocardial infarction
ENS: National health survey of Chile
CI: Confidence interval
NIH: National institute of health USA
IDF: International diabetes federation
